# Correlation of Increased Soluble Tumor Necrosis Factor Receptor 1 with Mortality and Dependence on Treatment in Non-Small-Cell Lung Cancer Patients: A Longitudinal Cohort Study

**DOI:** 10.3390/cancers16030525

**Published:** 2024-01-26

**Authors:** Lamiaa Hassan, Ahmed Bedir, Frank Bernhard Kraus, Christian Ostheimer, Dirk Vordermark, Rafael Mikolajczyk, Barbara Seliger, Daniel Medenwald

**Affiliations:** 1 Institute of Medical Epidemiology, Biometrics, and Informatics, Interdisciplinary Center for Health Sciences, Medical Faculty of the Martin Luther University Halle-Wittenberg, 06112 Halle (Saale), Germanyrafael.mikolajczyk@uk-halle.de (R.M.); 2 Department of Radiation Oncology, Health Services Research Group, University Hospital Halle (Saale), 06120 Halle (Saale), Germanydirk.vordermark@uk-halle.de (D.V.); 3 Department of Laboratory Medicine, Unit II LM-CC, University Hospital Halle (Saale), 06120 Halle (Saale), Germany; 4 Department of Radiation Oncology, University Hospital Halle (Saale), 06120 Halle (Saale), Germany; 5 Medical Faculty, Martin Luther University Halle-Wittenberg, 06120 Halle (Saale), Germany; 6 Institute for Translational Immunology, Brandenburg Medical School “Theodor Fontane”, 16816 Brandenburg, Germany; 7 Fraunhofer Institute for Cell Therapy and Immunology, 04103 Leipzig, Germany

**Keywords:** soluble TNF-receptor 1 (sTNF-R1), non-small-cell lung cancer (NSCLC), survival, inflammation, stereotactic body radiation therapy (SBRT)

## Abstract

**Simple Summary:**

This longitudinal cohort study investigates the role of soluble Tumor Necrosis Factor Receptor 1 (sTNF-R1) in non-small-cell lung cancer (NSCLC) patients. Serum sTNF-R1 levels were measured in 134 NSCLC patients before, during, and after treatment at the Medical Faculty of Martin Luther University Halle-Wittenberg between 2017 and 2019. This study reveals that baseline sTNF-R1 levels were higher in NSCLC patients compared to the general population, and they exhibited a linear increase over time. Importantly, individual changes in sTNF-R1 levels during and after treatment were found to be strongly associated with the risk of mortality, highlighting the potential of the sTNF-R1 trajectory as a valuable prognostic marker in NSCLC.

**Abstract:**

Background: Tumor necrosis factor (TNF) is a multipotent cytokine involved in inflammation and anti-tumor activity. TNF-α exerts its function upon binding to TNF-receptor 1 (TNF-R1) and TNF-receptor 2 (TNF-R2). This study investigates the relationship of soluble (s) TNF-R1 levels in non-small-cell lung cancer (NSCLC) patients with treatment and overall survival. Methods: In total, 134 NSCLC patients treated at the Medical Faculty of Martin Luther University Halle-Wittenberg between 2017 and 2019 were included in this study. Serum levels of sTNF-R1 were measured via ELISA at baseline and during and after treatment. A linear mixed-effects model was used to assess sTNF-R1 changes over time. Linear regression was applied to investigate the association between clinical characteristics and changes in sTNF-R1. Cox regression models were used to estimate associations with overall mortality. Results: The estimated average sTNFR-1 at baseline was 2091.71 pg/mL, with a change of 6.19 pg/mL per day. Cox models revealed that the individual change in sTNF-R1 was more strongly associated with mortality than its baseline value, especially after adjusting for covariates. Conclusions: This study provides evidence that the individual change in sTNF-R1 levels during and after treatment were associated with the risk of mortality, suggesting the use of the sTNF-R1 trajectory as a prognostic marker.

## 1. Introduction

Lung cancer (LC) is the leading cause of cancer-related deaths worldwide, with 64,804 new cases and 50,282 deaths in Germany alone in 2020 [1]. Non-small-cell lung cancer (NSCLC), a subtype of LC, accounts for approximately 85% of all cases. Despite progress in diagnosis and treatment methods, the 5-year survival rate for NSCLC stands at 15% [2]. Recent studies have highlighted the detrimental role that chronic inflammation plays with regard to the survival of LC patients [3,4,5]. Tumor necrosis factor alpha (TNF-α) is a highly versatile, multifunctional cytokine that is mainly produced by macrophages but also by mast cells, lymphocytes, endothelial cells, and fibroblasts [6]. It has been shown to orchestrate inflammation, regulate immune responses, and exhibit anti-tumor activity through a complex network of interactions that regulate cell apoptosis, activation, recruitment, and differentiation [7,8,9,10]. The physiological role of TNF-α can vary depending on its concentration and the specific cellular context. In some cases, TNF-α can promote tumor growth, while in others, it can induce tumor cell death [11,12]. Despite extensive research, the full range of TNF-α activities is still not completely understood [13]. TNF-α binds to the two distinct cell surface receptors TNF-receptor 1 (TNF-R1) and TNF-receptor 2 (TNF-R2), which are closely related but differ in their cellular distribution and effects on cell function, thereby leading to the induction of distinct inflammatory signaling pathways [14]. TNF-R1 activation induces both pro-inflammatory cell proliferation and cell apoptosis, while TNF-R2 primarily promotes cell proliferation without inducing apoptosis. This suggests that TNF-R1 is the more relevant receptor for the anti-tumor activity of TNF-α [15]. Furthermore, soluble forms of TNF-Rs (sTNF-Rs) exist, which are mediated by shedding, thereby inhibiting TNF-α. Consequently, the sTNF-Rs play a major role in regulating bioactive TNF-α levels [16]. Both sTNF-R1 and sTNF-R2 are found in the vascular circulation and modulate the activity of TNF-α in a physiological manner, but sTNF-Rs are often elevated in the serum/plasma of tumor patients. A recent meta-analysis of circulating TNF-R suggested its potential as a diagnostic biomarker [17]. Subsequent studies have shown that both elevated levels of TNF-α and TNF-Rs were related to a higher risk of endometrial cancer [18]. Moreover, ovarian cancer patients were found to have higher serum levels of TNF and sTNF-R compared to non-cancer subjects [19]. In addition, TNF-α and its receptors are reported to be broadly expressed in LC, and high TNF-α levels have been associated with primary and secondary resistance to targeted therapies in this disease [20,21,22]. While correlations between sTNF-R levels and the clinical stage, risk of initiation, and progression of various cancers have been documented [23], there exist limited data on sTNF-R levels in NSCLC and their behavior during treatment. Thus, this study aims to (i) compare sTNF-R levels at baseline with a matched sample from the general population and (ii) investigate the relationship of sTNF-R1 levels in NSCLC patients with treatment and overall survival.

## 2. Materials and Methods

### 2.1. Study Population

The current study included 134 NSCLC patients treated at the Radiation Oncology Department, Medical Faculty of the Martin Luther University Halle-Wittenberg, Halle, Germany, from 2017 to 2019. The inclusion criteria for this study required that participants were at least 18 years old, had provided signed informed consent, had a histopathologically confirmed diagnosis of NSCLC, and were indicated for either curative- or palliative-intent chemoradiotherapy. Participants were excluded from the study if they had received previous radiotherapy or had a secondary cancer within 5 years prior to study enrollment. These criteria were determined to ensure that the study samples were representative of NSCLC patients who are appropriate candidates for chemoradiotherapy. Matched samples from the CARdiovascular Living and Ageing (CARLA) cohort study, which has been described elsewhere, served as controls [24,25]. Briefly, the CARLA study recruited 1779 subjects (812 women and 967 men) aged 45–83 years from the general population in 2002. Notably, the CARLA control group consisted of individuals without a history of cancer. Furthermore, only individuals who had complete data on sTNF-R1 at baseline were included. All patients and participants in the CARLA cohort provided informed consent.

### 2.2. Determination of sTNF-R1 and CRP Levels

The sTNF-R1 levels were measured in the sera of NSCLC patients before, during, and after treatment, and analyses were performed at the Central Laboratory at the University Hospital of Halle (Saale). The measurement of sTNF-R1 concentrations was carried out using the Human sTNF-R1/TNFSF1A Immunoassay Quantikine® ELISA (R&D Systems, Minneapolis, MN, USA) on an Epoch 2 Microplate Spectrophotometer (BioTek, Bad Friedrichshall, Germany). sTNF-R1 concentrations were determined in duplicate, with standard quality controls (Quantikine Immunoassay Control Group; R&D Systems) and calibrations for each used microplate according to the recommendations of the manufacturer. C-reactive protein (CRP) was measured using a particle-enhanced immunoturbidimetric assay (CRP4, Roche Diagnostics, Rotkreuz, Switzerland) on a Roche cobas c701 analyzer integrated with a fully automated Roche Cobas 8000 platform according to the manufacturer’s instructions.

### 2.3. Outcome

The primary patient outcome measured was overall survival (OS), which was computed from the date of cancer diagnosis to the date of death from any cause. Alive patients were right-censored at the date of the last vital status assessment. To ascertain the vital status of all patients, we first consulted medical records. For those patients whose vital status could not be confirmed through medical records, we obtained the necessary information from the registration office.

### 2.4. Covariates

The dataset contained information on date of birth, sex, date of cancer diagnosis, tumor grading and histology, tumor–node–metastasis (TNM) stage, gross tumor volume (GTV), CRP, cause and date of death, and treatment information. The tumor stage at diagnosis was categorized into four groups based on the TNM cancer staging system. Treatment information included details on the administered radiation doses (equivalent dose in 2 gray (Gy) fractions (EQD2) and planning target volume (PTV) dose (Gy)).

### 2.5. Statistical Analysis

General descriptive statistics were calculated for the baseline characteristics of the sample. Continuous variables were displayed as means and standard deviations. Categorical variables were displayed as numbers and percentages. NSCLC patients were matched with participants from the CARLA cohort according to age, sex, BMI, CRP, and CRE using the inverse propensity score weighting method (MatchIT package in R) [26]. To investigate the mean rate of sTNF-R1 change per day among NSCLC patients during treatment, a linear mixed-effects model with a random intercept and a random slope using the R package “lme4” was employed [27]. This model choice was driven by the need to capture both the individual variability in baseline sTNF-R1 levels (random intercept) and the rate of change in these levels over time for each patient (random slope), thereby acknowledging the unique progression of NSCLC in each individual. The estimates for the random intercept and random slope were then standardized, providing a more intuitive understanding of these metrics. The association of baseline characteristics (standardized random intercept, age, sex, EQD2, stage at diagnosis, stereotactic body radiation therapy (SBRT), chemotherapy, and PTV) with the standardized slope-change measurements was investigated using a linear model. This approach not only accounts for individual patient differences but also offers insights into the clinical significance of these findings. For instance, a higher random intercept might suggest a more aggressive baseline disease state, while a steeper random slope could indicate a rapid progression or response to treatment, thus providing valuable information for patient management and prognosis. Cox proportional hazards models were used to estimate the hazard ratio (HR) for mortality with a 95% confidence interval (CI), with the standardized random intercept and slope of sTNF-R1 as predictors. Various models were fitted and compared: The base model included adjustments for age and sex. In the second model, EQD2, TNM stage, chemotherapy, and planning target volume dose were additionally included, while in the third model, CRP at baseline was also included. These sequential adjustments in our models were designed to control for a range of variables, ensuring that the observed associations are specifically related to sTNF-R1 levels and not confounded by the diverse patient characteristics within our cohort. All analyses were conducted using the R statistical software version 3.2.3 [28].

## 3. Results

In total, 134 patients diagnosed with NSCLC between 2017 and 2019 were included in this study (Table 1). From the follow-up until the end of 2020, 54 (41%) patients died. The mean age at diagnosis for all patients was 68.2 ± 10 years (range 42.3–88.1). The majority of the NSCLC cases were diagnosed at stages IV and III (39% and 36%, respectively), with a gross tumor volume average of 106.3 cm³. Sixty-five (52%) patients received adjuvant chemotherapy, while 32% received SBRT.

**Figure 1:**
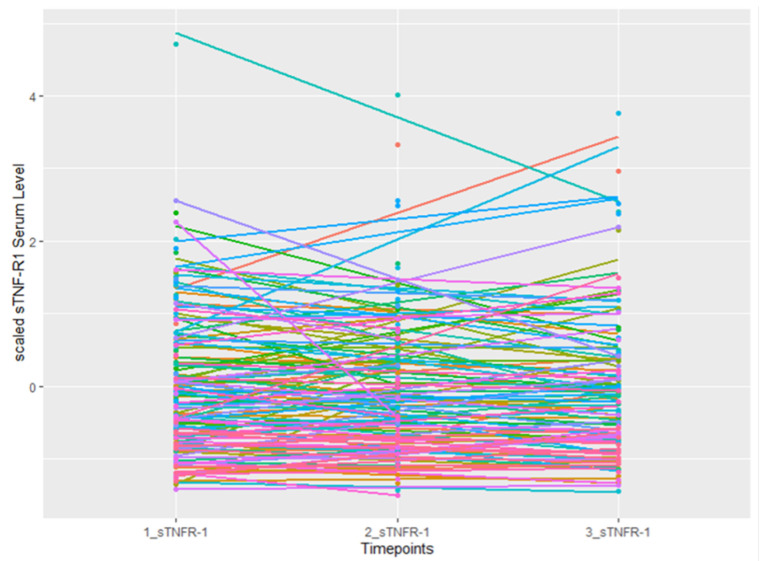
Temporal changes in scaled sTNFR1 serum levels in 134 NSCLC patients.

The sTNF-R1 serum levels prior to treatment averaged 2081.62 pg/mL. The average of sTNF-R1 serum levels in the samples increased linearly for the second and third time points (2228.24 pg/mL and 2298.40 pg/mL, respectively) (Figure 1).

**Figure 2:**
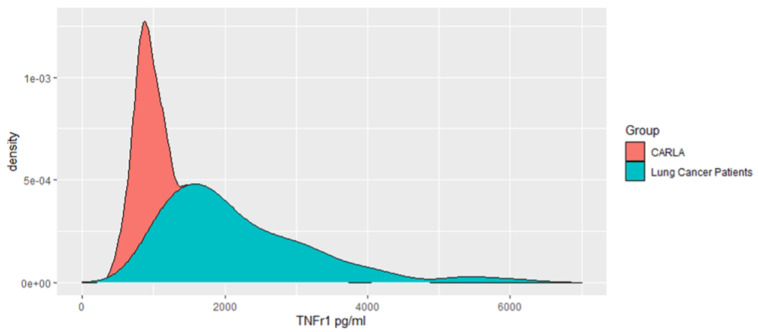
sTNFR1 serum levels in 134 NSCLC patients and 304 CARLA participants.

In comparison to CARLA participants (n = 303, 1237.97 pg/mL, 95% CI: 452.3–4693.7 pg/mL), the baseline (pre-treatment) serum levels of sTNF-R1 in NSCLC patients were remarkably higher and had a wider range (2196.51 pg/mL, 95% CI: 879–6066 pg/mL) (Figure 2).

The linear mixed-effects model estimated the mean intercept, which represented the estimated average of sTNFR-1 level (2091.71 pg/mL; 1964.43–2219.04) at the first examination across all patients, aligning closely with the observed average sTNFR-1 level at the first examination. The mean change in serum sTNF-R1 levels across all patients for each additional day was 6.19 pg/mL (2.52–10.15). The standard deviations were approximately 715.99 (pg/mL) for the random intercept and 4.21 (pg/mL/day) for the random slope, indicating variability in the initial sTNFR-1 levels and a relatively low variability in the rate of change over time among patients, respectively. The covariance between the random intercept and slope (9322 (pg/mL)^2^) suggests a relationship between the baseline sTNFR-1 level and its rate of change among patients, emphasizing that this variation is not solely attributed to baseline levels. A unit change in the standardized random intercept corresponds to a difference of about 715.99 pg/mL from the average baseline sTNF-R1, while a unit change in the standardized random slope indicates a rate of change that differs by about 4.21 pg/mL per day from the average rate of change across all patients. Regarding the baseline characteristics included in the linear regression, only SBRT was significantly associated with the individual change in sTNF-R1 (ß = 0.94 SD units for those who received SBRT (0.33–1.55), respectively (Table 2).

The crude Cox regression model (Model 1) showed an initial value of sTNF-R1 associated with all-cause mortality (hazard ratio (HR) 1.38 per one standard deviation, 95% CI: 1.1–1.8) (Table 3). This suggests that higher baseline sTNF-R1 levels are slightly more associated with an increased risk of mortality compared to the association between the individual change in the slope of sTNF-R1 measurements and all-cause mortality (HR 1.22 per one standard deviation, 95% CI: 0.9–1.7). After adjustment for age, sex, EQD2, TNM stage, chemotherapy, SBRT, PTV and GTBV in Model 2, the association between slope and mortality increased to 2.60 (95% CI: 1.4–4.7), while it decreased for the baseline measurement (HR 1.16, 95% CI: 0.8–1.5), indicating that the rate of change in sTNF-R1 levels is a stronger predictor of mortality than baseline levels alone. The addition of baseline CRP in Model 3 did not meaningfully alter the HR for either the intercept or slope. This stability in the results suggests that the association between sTNF-R1 levels and mortality is not confounded by baseline CRP levels, underscoring the independent predictive value of sTNF-R1 measurements.

## 4. Discussion

Increased TNF-α and TNF-R expression has been implicated in various cancers, including NSCLC, indicating a significant role for these molecules in the disease’s development and progression [22,29]. Additionally, elevated levels of circulating sTNF-R1 in serum samples from NSCLC patients have been proposed to have prognostic significance [23]. In our study, we noted a potential association between sTNF-R1 levels and all-cause mortality. It was evident that NSCLC patients had higher sTNF-R1 levels compared to matched controls from the general population, indicating a notable physiological difference. Furthermore, the rate of change in sTNF-R1 levels exhibited a stronger association with all-cause mortality than the baseline sTNF-R1 levels after adjustments for patient and tumor characteristics, as well as the treatment received. These findings suggest that TNF-R1 may play a crucial role in NSCLC development and progression. Our findings are consistent with previous studies reporting elevated levels of sTNF-R1 in patients with solid tumors compared to controls. For example, higher serum TNF-R1 levels were found in patients with endometrial cancer [18], colorectal cancer [30], pancreatic cancer [31], and glioblastoma [32] when compared to healthy controls. Since TNFR-1 is involved in inflammation and the immune response and its overproduction has been linked to the development and progression of various cancers, including LC, our findings suggest that elevated sTNF-R1 levels may be a prognostic marker [21]. Furthermore, our results also showed that the standardized change in sTNF-R1 levels over time, rather than exclusively during treatment, had a more robust association with mortality than initial levels measured at baseline after adjusting for patient and tumor characteristics and the treatment received. While these findings suggest that monitoring sTNF-R1-level changes might be informative in understanding patient outcomes, it is essential to note that there is variability in these changes over time, albeit less pronounced than the variability in baseline levels. Therefore, while changes in sTNF-R1 levels could potentially aid in patient outcome assessment, further research is needed to establish their predictive utility more definitively. To our knowledge, this study is the first to investigate the effect of sTNF-R1 changes in cancer patients receiving radiotherapy on OS. In a related context, Siva and co-authors explored the kinetics of radiation-induced plasma inflammatory cytokines in NSCLC patients. Their findings revealed that early changes in specific cytokines, such as IP-10 (interferon gamma-induced protein-10) and IL-6 (interleukin-6), were associated with a higher grade of toxicity, emphasizing the potential of cytokine measurements during radiotherapy as predictors of lung toxicity [33].

Our study also identified that SBRT, commonly administered in early-stage NSCLC, was significantly associated with the individual change in sTNF-R1 across time points. This finding suggests a significant role for SBRT in influencing immune responses, potentially leading to the regulation of sTNF-R1 levels. While the underlying mechanisms of this association are not fully understood and warrant further investigation [34,35], it is evident that SBRT, even more so than the planning target volume (PTV) dose, plays a crucial role in modulating this immune marker. As the disease progresses, the immune system’s response to specific antigens diminishes [36]. Elevated cytokines, like sTNF-R1, could signify the immune system’s counteraction to cancer cells. However, certain cytokines may decrease due to the immune system’s regulation and cancer cells’ attempt to suppress immune activity. The variability in cytokine levels, potentially due to tumor responses during radiation therapy, suggests that cytokines could serve as indicators of the disease response to RT. While our analysis included various treatments like radiotherapy and chemotherapy, the specific impact of immunotherapy was not examined, as our sTNF-R-level measurements were conducted just before, during, and immediately after the completion of the patients’ chemoradiotherapy sessions. Typically, in accordance with the treatment guidelines at the time of our study, immunotherapy was administered after the conclusion of chemoradiotherapy. Consequently, this occurred beyond the scope of our data collection time frame. Despite this, the role of immunotherapy in NSCLC, especially treatments targeting the PD1/PDL1 pathway in advanced stages, is becoming increasingly significant. Such therapies have the potential to alter the immune response, potentially affecting levels of inflammatory cytokines like TNF. This could have substantial implications for patient outcomes and biomarker dynamics. Therefore, the absence of immunotherapy data in our study is a notable limitation. Considering current treatment guidelines, future research should aim to encompass immunotherapy data, both when administered post-chemoradiotherapy and concurrently, to thoroughly understand its influence on inflammatory processes and TNF-related responses in NSCLC patients.

A strength of our study is the longitudinal design, which allowed us to assess the association between sTNF-R1 levels and mortality over time by determining the sTNF-R1 levels before, during, and after treatment. This is particularly important in cancer patients, as the disease and treatment may lead to changes in biomarker levels over time. Therefore, our findings provide valuable insights into the dynamic changes in sTNF-R1 levels during the course of treatment and their association with mortality. The relatively small number of patients recruited in our study primarily affects its statistical power, potentially limiting our ability to detect certain effects or associations. This aspect should be considered when interpreting the results, as it may influence the robustness of our findings. In addition, the potential mechanism of action underlying the association between TNFR-1 and all-cause mortality was not investigated. The significantly lower sTNF-R levels prior to treatment might be associated with changes in systemic immune suppression involving different cellular and cytokine compartments [32]. Future studies should explore the biological mechanisms by which TNFR-1 affects all-cause mortality in NSCLC patients.

## 5. Conclusions

In conclusion, our study provides a more detailed view of the role of the sTNFR-1 level in NSCLC patients and its association with all-cause mortality. Our findings suggest that elevated levels of sTNF-R1 in NSCLC patients may be associated with a higher risk of mortality and that changes in sTNF-R1 levels over time may be a more prognostic biomarker of mortality than baseline levels. The prognostic value of sTNF-R1 as a biomarker, however, should be interpreted with consideration of the observed variability. Additionally, our analysis indicates a notable link between SBRT, typically administered in early-stage NSCLC, and sTNF-R1 level changes. This effect appears to be more pronounced than that of the PTV dose, suggesting a distinct role for SBRT in influencing these levels. This observation underscores the need to further investigate how different treatment modalities, particularly in different stages of NSCLC, impact patient prognosis. Future research should not only aim to validate these findings but also explore the complex interplay between sTNF-R1 levels, treatment approaches, other biomarkers, and clinical factors to enhance our understanding of NSCLC prognosis.

## Figures and Tables

**Table 1:** Baseline characteristics of lung cancer patients diagnosed with NSCLC from 2017 to 2019.

Patient Characteristics	
Age (years), mean	68.18 (10)
Sex (female)	60 (45%)
1st sTNF-RI (pg/mL), mean	2081.62 (844)
CRP (mg/L), mean	53.8 (68)
Adjuvant chemotherapy	65 (52%)
Stereotactic body radiation therapy (SBRT)	33 (32%)
Type of treatment (palliative)	48 (36%)
TNM stage I	19 (15%)
TNM stage II	13 (10%)
TNM stage III	47 (36%)
TNM stage IV	51 (39%)
Gross tumor volume (GTV) (ccm)	106.3 (266)
Planning target volume (PTV) dose (Gy)	62.0 (24)
2nd sTNF-RI (pg/mL), mean	2228.24 (917)
3rd sTNF-RI (pg/mL), mean	2298.40 (1026)
Status (Dead)	54 (41%)

Note: SD represents the standard deviation. Percentages are shown in parentheses next to the corresponding data.

**Table 2:** Association of baseline characteristics with the slope of three sTNF-RI measurements using linear regression.

	Adjusted β-Estimates * (95% CI)	*p*-Value
Intercept	0.211 (−1.754–2.305)	0.79
Standardized mean of sTNFR-1 at first measurement	−0.0460 (−0.3–0.2)	0.67
Age (in years)	0.008 (−0.013–0.029)	0.45
Sex (female)	0.0988 (−343–0.555)	0.67
Chemotherapy vs no chemotherapy	0.203 (−0.22–0.63)	0.34
TNM stage II vs. I	−0.17 (−1.040–0.69)	0.69
TNM stage III vs. I	−0.18 (−0.98–0.62)	0.65
TNM stage IV vs. I	−0.29 (−1.11–0.54)	0.49
Log (CRP) (mg/L)	−0.01 (−0.15–0.12)	0.84
Stereotactic body radiation therapy (SBRT) vs. no SBRT	0.95 (0.33–1.58)	0.003

* Adjusted ß-estimates indicate the independent effect of each variable on the rate of change in sTNF-R1 levels, considering other model factors. Positive ß-estimates suggest an increased rate of change, and negative ones indicate a decreased rate of change.

**Table 3:** Association of intercept and slope of three sTNF-RI measurements with all-cause mortality estimated by using Cox regression models.

	Adjusted HR (95% CI) Model 1	Adjusted HR (95% CI) Model 2	Adjusted HR (95% CI) Model 3 with CRP
Standardized baseline sTNF-RI (pg/mL)	1.38 (1.1–1.8)	1.16 (0.8–1.5)	1.16 (0.9–1.6)
Standardized change in sTNF-RI (pg/mL/day)	1.22 (0.9–1.7)	2.60 (1.4–4.7)	2.60 (1.4–4.7)

Intercept and slope were included in each model. Model 1 is adjusted for age and sex. Model 2 is adjusted for covariates of Model 1 and additionally adjusted for EQD2c, TNM stage, stereotactic body radiation therapy, chemotherapy, and planning target volume dose. Model 3 is adjusted for all covariates of Model 2 and additionally adjusted for CRP. HR, hazard ratio; CI, confidence interval.

## Data Availability

The datasets generated and/or analyzed during the current study are not publicly available due to the presence of sensitive and confidential patient information but are available from the corresponding author on reasonable request.

## Abbreviations

The following abbreviations are used in this manuscript:

BMI	Body Mass Index
CARLA	CARdiovascular Living and Ageing
CI	Confidence Interval
CRP	C-reactive Protein
EQD2	Equivalent Dose in 2 Gray
GTV	Gross Tumor Volume
HR	Hazard Ratio
LC	Lung Cancer
NSCLC	Non-Small-Cell Lung Cancer
OS	Overall Survival
PTV	Planning Target Volume
IP-10	Interferon Gamma-induced Protein-10
IL-6	Interleukin-6
SBRT	Stereotactic Body Radiation Therapy
sTNF-R	Soluble TNF Receptor
TNF	Tumor Necrosis Factor
TNF-R	Tumor Necrosis Factor Receptor
TNM	Tumor Node Metastasis
